# Supplementary material for: Single Cell Transcriptomic and Chromatin Profiles Suggest Layer Vb Is the Only Layer With Shared Excitatory Cell Types in the Medial and Lateral Entorhinal Cortex
Source: Front Neural Circuits. 2022 Jan 26;15:806154. doi: 10.3389/fncir.2021.806154 (PMC8826650; doi:10.3389/fncir.2021.806154)
Supplement: Supplementary file 14 [file Data_Sheet_1.ZIP › 7/knownResults.html]

7 - Homer Known Motif Enrichment Results


# Homer Known Motif Enrichment Results (7)

Homer *de novo* Motif Results  
Gene Ontology Enrichment Results  
Known Motif Enrichment Results (txt file)  
Total Target Sequences = 1197, Total Background Sequences = 47978

|  |  |  |  |  |  |  |  |  |  |  |  |
| --- | --- | --- | --- | --- | --- | --- | --- | --- | --- | --- | --- |
| Rank | Motif | Name | P-value | log P-pvalue | q-value (Benjamini) | # Target Sequences with Motif | % of Targets Sequences with Motif | # Background Sequences with Motif | % of Background Sequences with Motif | Motif File | SVG |
| 1 | T C G A T G A C G T A C C G T A C A G T T G A C A C G T A C T G A G C T A G C T | NeuroG2(bHLH)/Fibroblast-NeuroG2-ChIP-Seq(GSE75910)/Homer | 1e-153 | -3.527e+02 | 0.0000 | 684.0 | 57.14% | 10488.0 | 21.86% | motif file (matrix) | svg |
| 2 | C T G A T G A C T G A C C G T A A C G T T G A C A G C T C T A G A C G T G A C T | Olig2(bHLH)/Neuron-Olig2-ChIP-Seq(GSE30882)/Homer | 1e-144 | -3.334e+02 | 0.0000 | 790.0 | 66.00% | 14371.8 | 29.95% | motif file (matrix) | svg |
| 3 | C T A G T C G A T G A C A G T C C G T A A C T G G T A C A C G T A C T G A C T G | BHLHA15(bHLH)/NIH3T3-BHLHB8.HA-ChIP-Seq(GSE119782)/Homer | 1e-143 | -3.294e+02 | 0.0000 | 622.0 | 51.96% | 9106.2 | 18.98% | motif file (matrix) | svg |
| 4 | T C A G A C G T T C G A T A G C A G T C C G T A A C T G G T A C A C G T A C T G A T C G A G T C | Atoh1(bHLH)/Cerebellum-Atoh1-ChIP-Seq(GSE22111)/Homer | 1e-138 | -3.195e+02 | 0.0000 | 506.0 | 42.27% | 6195.6 | 12.91% | motif file (matrix) | svg |
| 5 | T G A C A G T C C G T A A C T G G T A C A C G T A C T G A C G T G A C T G A T C | Twist2(bHLH)/Myoblast-Twist2.Ty1-ChIP-Seq(GSE127998)/Homer | 1e-135 | -3.129e+02 | 0.0000 | 705.0 | 58.90% | 11969.4 | 24.95% | motif file (matrix) | svg |
| 6 | T A C G T G C A A G T C C G T A A C G T T G A C A C G T A C T G A C T G G C A T | TCF4(bHLH)/SHSY5Y-TCF4-ChIP-Seq(GSE96915)/Homer | 1e-121 | -2.789e+02 | 0.0000 | 636.0 | 53.13% | 10546.6 | 21.98% | motif file (matrix) | svg |
| 7 | T C A G T G A C G T A C C G T A A C G T T G A C A C G T T C A G A G C T G A C T | NeuroD1(bHLH)/Islet-NeuroD1-ChIP-Seq(GSE30298)/Homer | 1e-114 | -2.632e+02 | 0.0000 | 409.0 | 34.17% | 4744.9 | 9.89% | motif file (matrix) | svg |
| 8 | C T A G A G T C T A C G T A C G T G A C C G T A A C T G T A G C G C A T C A T G A T G C A G C T | Ascl1(bHLH)/NeuralTubes-Ascl1-ChIP-Seq(GSE55840)/Homer | 1e-74 | -1.714e+02 | 0.0000 | 462.0 | 38.60% | 7884.6 | 16.43% | motif file (matrix) | svg |
| 9 | T C G A A G T C C G T A A T C G T A G C A C G T A C T G A G C T A C G T A G T C | Ptf1a(bHLH)/Panc1-Ptf1a-ChIP-Seq(GSE47459)/Homer | 1e-71 | -1.652e+02 | 0.0000 | 693.0 | 57.89% | 15608.3 | 32.53% | motif file (matrix) | svg |
| 10 | C T G A T C A G G T A C G C T A A C T G T G A C G C A T C A T G | SCL(bHLH)/HPC7-Scl-ChIP-Seq(GSE13511)/Homer | 1e-66 | -1.534e+02 | 0.0000 | 958.0 | 80.03% | 26993.3 | 56.26% | motif file (matrix) | svg |
| 11 | C G A T T G C A T G C A G A T C C G T A A C T G T G A C G A C T C A T G A C T G | Tcf21(bHLH)/ArterySmoothMuscle-Tcf21-ChIP-Seq(GSE61369)/Homer | 1e-53 | -1.229e+02 | 0.0000 | 319.0 | 26.65% | 5102.6 | 10.64% | motif file (matrix) | svg |
| 12 | T G A C A T G C C G T A A T C G A T G C C A G T C A T G A C T G A G T C G T A C | HEB(bHLH)/mES-Heb-ChIP-Seq(GSE53233)/Homer | 1e-44 | -1.028e+02 | 0.0000 | 460.0 | 38.43% | 9876.8 | 20.59% | motif file (matrix) | svg |
| 13 | A G C T G T C A C G T A A C G T A C G T C T G A T C A G A T G C | Lhx2(Homeobox)/HFSC-Lhx2-ChIP-Seq(GSE48068)/Homer | 1e-41 | -9.491e+01 | 0.0000 | 459.0 | 38.35% | 10141.4 | 21.14% | motif file (matrix) | svg |
| 14 | A T C G A T C G A T G C G A C T T C G A C G T A G C A T A G C T C T G A T A C G | DLX2(Homeobox)/BasalGanglia-Dlx2-ChIP-seq(GSE124936)/Homer | 1e-40 | -9.381e+01 | 0.0000 | 583.0 | 48.71% | 14438.8 | 30.09% | motif file (matrix) | svg |
| 15 | A T G C T A C G A G C T T G C A C G T A C G A T A C G T C T G A | DLX5(Homeobox)/BasalGanglia-Dlx5-ChIP-seq(GSE124936)/Homer | 1e-39 | -9.097e+01 | 0.0000 | 371.0 | 30.99% | 7504.2 | 15.64% | motif file (matrix) | svg |
| 16 | A T C G A T G C A G T C T A G C G A C T T C G A G C T A G C A T A G C T C T G A | DLX1(Homeobox)/BasalGanglia-Dlx1-ChIP-seq(GSE124936)/Homer | 1e-38 | -8.918e+01 | 0.0000 | 524.0 | 43.78% | 12566.1 | 26.19% | motif file (matrix) | svg |
| 17 | T C A G C T A G A T G C G A C T T G C A C G T A A G C T A C G T C T G A T A C G | En1(Homeobox)/SUM149-EN1-ChIP-Seq(GSE120957)/Homer | 1e-35 | -8.180e+01 | 0.0000 | 620.0 | 51.80% | 16364.6 | 34.11% | motif file (matrix) | svg |
| 18 | G T C A T G C A G C T A A G T C C G T A A C T G T G A C G C A T T C A G C A G T | Ap4(bHLH)/AML-Tfap4-ChIP-Seq(GSE45738)/Homer | 1e-34 | -7.891e+01 | 0.0000 | 341.0 | 28.49% | 7013.1 | 14.62% | motif file (matrix) | svg |
| 19 | A T G C T A C G A G T C G A C T T G C A C T G A G A C T A C G T C T G A T C A G | LHX9(Homeobox)/Hct116-LHX9.V5-ChIP-Seq(GSE116822)/Homer | 1e-32 | -7.579e+01 | 0.0000 | 511.0 | 42.69% | 12728.1 | 26.53% | motif file (matrix) | svg |
| 20 | C G T A C A T G A G T C G A C T T G C A C G T A A C G T A C G T C T G A T C A G | Lhx1(Homeobox)/EmbryoCarcinoma-Lhx1-ChIP-Seq(GSE70957)/Homer | 1e-32 | -7.499e+01 | 0.0000 | 459.0 | 38.35% | 10975.9 | 22.88% | motif file (matrix) | svg |
| 21 | T C G A C A G T A C T G A G C T C G T A C G T A A C G T A C G T C T G A T A G C | Dlx3(Homeobox)/Kerainocytes-Dlx3-ChIP-Seq(GSE89884)/Homer | 1e-31 | -7.250e+01 | 0.0000 | 314.0 | 26.23% | 6425.8 | 13.39% | motif file (matrix) | svg |
| 22 | A C G T T C G A T C G A A G T C G T C A T A C G A T G C A C G T A C T G A G C T | Myf5(bHLH)/GM-Myf5-ChIP-Seq(GSE24852)/Homer | 1e-29 | -6.693e+01 | 0.0000 | 210.0 | 17.54% | 3629.8 | 7.57% | motif file (matrix) | svg |
| 23 | A C T G C A G T A C G T C G T A C G T A A C G T A C T G C T G A | Nkx6.1(Homeobox)/Islet-Nkx6.1-ChIP-Seq(GSE40975)/Homer | 1e-28 | -6.675e+01 | 0.0000 | 782.0 | 65.33% | 23572.4 | 49.13% | motif file (matrix) | svg |
| 24 | C G T A T G A C T C G A A G T C C G T A A T C G A T G C A C G T A C T G A G T C | E2A(bHLH)/proBcell-E2A-ChIP-Seq(GSE21978)/Homer | 1e-28 | -6.513e+01 | 0.0000 | 326.0 | 27.23% | 7073.0 | 14.74% | motif file (matrix) | svg |
| 25 | T C G A A G T C C G T A A T C G A T G C C G A T A C T G A G T C A G C T A C T G | Tcf12(bHLH)/GM12878-Tcf12-ChIP-Seq(GSE32465)/Homer | 1e-27 | -6.247e+01 | 0.0000 | 234.0 | 19.55% | 4426.9 | 9.23% | motif file (matrix) | svg |
| 26 | T C G A T C G A A G T C C G T A C T A G T A G C A C G T A C T G | MyoG(bHLH)/C2C12-MyoG-ChIP-Seq(GSE36024)/Homer | 1e-25 | -5.900e+01 | 0.0000 | 258.0 | 21.55% | 5241.9 | 10.93% | motif file (matrix) | svg |
| 27 | T A C G T A C G C T A G T C A G A G T C C G T A A T C G A T G C A C G T A C T G A G T C G A C T | Ascl2(bHLH)/ESC-Ascl2-ChIP-Seq(GSE97712)/Homer | 1e-23 | -5.503e+01 | 0.0000 | 255.0 | 21.30% | 5300.3 | 11.05% | motif file (matrix) | svg |
| 28 | C G T A C G T A A G C T G A C T T G C A G T C A A C G T A G C T C T G A T C A G | Lhx3(Homeobox)/Neuron-Lhx3-ChIP-Seq(GSE31456)/Homer | 1e-23 | -5.480e+01 | 0.0000 | 567.0 | 47.37% | 15898.4 | 33.14% | motif file (matrix) | svg |
| 29 | C T G A T C A G A G T C C G T A A T C G A T G C C G A T A C T G A G T C G A C T A T C G A G T C | MyoD(bHLH)/Myotube-MyoD-ChIP-Seq(GSE21614)/Homer | 1e-20 | -4.758e+01 | 0.0000 | 186.0 | 15.54% | 3560.3 | 7.42% | motif file (matrix) | svg |
| 30 | A G T C C G A T C T G A C G T A A C G T C A G T T C A G T G A C | Isl1(Homeobox)/Neuron-Isl1-ChIP-Seq(GSE31456)/Homer | 1e-18 | -4.313e+01 | 0.0000 | 546.0 | 45.61% | 15883.7 | 33.11% | motif file (matrix) | svg |
| 31 | C T G A C T G A C A T G A T C G A G C T A T C G G A C T C A T G C T G A G T C A | Tbr1(T-box)/Cortex-Tbr1-ChIP-Seq(GSE71384)/Homer | 1e-16 | -3.690e+01 | 0.0000 | 387.0 | 32.33% | 10544.9 | 21.98% | motif file (matrix) | svg |
| 32 | C T G A C T A G A T C G A G C T A C T G G A C T A G T C C T G A | Tbx5(T-box)/HL1-Tbx5.biotin-ChIP-Seq(GSE21529)/Homer | 1e-14 | -3.372e+01 | 0.0000 | 623.0 | 52.05% | 19558.0 | 40.76% | motif file (matrix) | svg |
| 33 | T G C A C T G A A T G C G T C A A C G T A T G C A C G T A C T G A C T G T G C A | ZBTB18(Zf)/HEK293-ZBTB18.GFP-ChIP-Seq(GSE58341)/Homer | 1e-14 | -3.263e+01 | 0.0000 | 154.0 | 12.87% | 3185.5 | 6.64% | motif file (matrix) | svg |
| 34 | A G T C A C G T A C T G A G C T A C G T A C G T G T C A A G T C | Foxo1(Forkhead)/RAW-Foxo1-ChIP-Seq(Fan\_et\_al.)/Homer | 1e-13 | -3.102e+01 | 0.0000 | 484.0 | 40.43% | 14484.6 | 30.19% | motif file (matrix) | svg |
| 35 | G C T A G A C T G A C T T G C A C G T A A G T C C G T A T A G C G A T C G A C T | Eomes(T-box)/H9-Eomes-ChIP-Seq(GSE26097)/Homer | 1e-12 | -2.912e+01 | 0.0000 | 528.0 | 44.11% | 16297.8 | 33.97% | motif file (matrix) | svg |
| 36 | C T G A C T A G A C T G G C A T C T A G G C A T A T C G C T G A C G T A G T C A | Tbx21(T-box)/GM12878-TBX21-ChIP-Seq(Encode)/Homer | 1e-12 | -2.862e+01 | 0.0000 | 270.0 | 22.56% | 7061.8 | 14.72% | motif file (matrix) | svg |
| 37 | G A C T C G A T C T G A G T C A G A C T C G A T T C G A C G T A G C T A G C T A T G A C G T A C C G T A A C T G T G C A C G A T A C T G A C G T | Pitx1:Ebox(Homeobox,bHLH)/Hindlimb-Pitx1-ChIP-Seq(GSE41591)/Homer | 1e-12 | -2.848e+01 | 0.0000 | 97.0 | 8.10% | 1734.3 | 3.61% | motif file (matrix) | svg |
| 38 | A G C T C A T G G C A T G A T C T G C A C T A G G A T C A C G T | Tgif2(Homeobox)/mES-Tgif2-ChIP-Seq(GSE55404)/Homer | 1e-12 | -2.798e+01 | 0.0000 | 700.0 | 58.48% | 23136.0 | 48.22% | motif file (matrix) | svg |
| 39 | C G A T C T A G A C G T G T C A C G T A C G T A A G T C C G T A | Foxo3(Forkhead)/U2OS-Foxo3-ChIP-Seq(E-MTAB-2701)/Homer | 1e-10 | -2.485e+01 | 0.0000 | 266.0 | 22.22% | 7175.0 | 14.95% | motif file (matrix) | svg |
| 40 | A T G C G T A C C G T A A G C T G C A T T A C G A G C T A G C T A G T C A G C T | Sox6(HMG)/Myotubes-Sox6-ChIP-Seq(GSE32627)/Homer | 1e-10 | -2.424e+01 | 0.0000 | 466.0 | 38.93% | 14400.7 | 30.01% | motif file (matrix) | svg |
| 41 | C G T A C T A G C A T G A G C T A C T G C G A T A T C G C G T A G T C A G T C A | Tbet(T-box)/CD8-Tbet-ChIP-Seq(GSE33802)/Homer | 1e-9 | -2.232e+01 | 0.0000 | 281.0 | 23.48% | 7874.0 | 16.41% | motif file (matrix) | svg |
| 42 | A G C T T G A C C G A T C G A T C T A G A C G T C A G T C A G T G C T A A G T C | FOXK1(Forkhead)/HEK293-FOXK1-ChIP-Seq(GSE51673)/Homer | 1e-9 | -2.075e+01 | 0.0000 | 343.0 | 28.65% | 10200.0 | 21.26% | motif file (matrix) | svg |
| 43 | T C A G T A C G T A G C A C G T A C T G C G A T A G T C C G T A T A C G A G T C | Meis1(Homeobox)/MastCells-Meis1-ChIP-Seq(GSE48085)/Homer | 1e-8 | -1.975e+01 | 0.0000 | 357.0 | 29.82% | 10798.0 | 22.51% | motif file (matrix) | svg |
| 44 | G A C T G C A T C T A G C G A T G A T C T C G A C A T G G A T C | Tgif1(Homeobox)/mES-Tgif1-ChIP-Seq(GSE55404)/Homer | 1e-8 | -1.968e+01 | 0.0000 | 665.0 | 55.56% | 22599.4 | 47.10% | motif file (matrix) | svg |
| 45 | A G C T A G T C A G T C A C G T C T A G A C G T A C G T A C G T C G T A A G T C G A T C C G T A | FOXP1(Forkhead)/H9-FOXP1-ChIP-Seq(GSE31006)/Homer | 1e-8 | -1.850e+01 | 0.0000 | 165.0 | 13.78% | 4225.9 | 8.81% | motif file (matrix) | svg |
| 46 | C T A G C T G A T C A G A T C G A G C T C A T G G A C T A G T C C T G A T G C A | Tbx6(T-box)/ESC-Tbx6-ChIP-Seq(GSE93524)/Homer | 1e-7 | -1.834e+01 | 0.0000 | 236.0 | 19.72% | 6624.8 | 13.81% | motif file (matrix) | svg |
| 47 | A G C T A C G T A C T G A T G C A G T C C G T A C T G A T A C G | NF1-halfsite(CTF)/LNCaP-NF1-ChIP-Seq(Unpublished)/Homer | 1e-7 | -1.831e+01 | 0.0000 | 320.0 | 26.73% | 9592.6 | 19.99% | motif file (matrix) | svg |
| 48 | C T A G T A C G G A T C G T A C G C T A A G C T A G C T G T C A T C G A T A G C | Nanog(Homeobox)/mES-Nanog-ChIP-Seq(GSE11724)/Homer | 1e-7 | -1.780e+01 | 0.0000 | 847.0 | 70.76% | 30308.3 | 63.17% | motif file (matrix) | svg |
| 49 | G A C T C T A G A T G C A G T C G T C A T A C G A T G C A T C G | HIC1(Zf)/Treg-ZBTB29-ChIP-Seq(GSE99889)/Homer | 1e-7 | -1.746e+01 | 0.0000 | 342.0 | 28.57% | 10470.7 | 21.82% | motif file (matrix) | svg |
| 50 | T G C A G T A C C G T A A T C G A C T G A C G T C T A G C G A T T C G A A G T C | ZEB1(Zf)/PDAC-ZEB1-ChIP-Seq(GSE64557)/Homer | 1e-7 | -1.724e+01 | 0.0000 | 329.0 | 27.49% | 10019.6 | 20.88% | motif file (matrix) | svg |
| 51 | C A T G A C T G C T A G T C G A T C G A T C G A T C G A T C A G T C A G T C A G T G A C T G A C C G T A A C T G T G C A C G A T A C T G | RBPJ:Ebox(?,bHLH)/Panc1-Rbpj1-ChIP-Seq(GSE47459)/Homer | 1e-6 | -1.589e+01 | 0.0000 | 81.0 | 6.77% | 1743.0 | 3.63% | motif file (matrix) | svg |
| 52 | A C T G C A T G T C G A A C G T A C T G C G T A A T C G A C G T G T A C C G T A G A C T A G T C | Fos(bZIP)/TSC-Fos-ChIP-Seq(GSE110950)/Homer | 1e-6 | -1.518e+01 | 0.0000 | 153.0 | 12.78% | 4050.6 | 8.44% | motif file (matrix) | svg |
| 53 | C A T G C T A G T C G A A C G T A C T G C G T A T A G C C G A T T G A C C G T A A G C T G A T C | Fra2(bZIP)/Striatum-Fra2-ChIP-Seq(GSE43429)/Homer | 1e-6 | -1.511e+01 | 0.0000 | 121.0 | 10.11% | 3013.6 | 6.28% | motif file (matrix) | svg |
| 54 | A G C T T C G A G T A C T C G A A T G C A T G C G C A T A T C G A G T C A G C T | Snail1(Zf)/LS174T-SNAIL1.HA-ChIP-Seq(GSE127183)/Homer | 1e-6 | -1.441e+01 | 0.0000 | 162.0 | 13.53% | 4405.2 | 9.18% | motif file (matrix) | svg |
| 55 | A C T G C T A G T C G A C G A T C A T G G C T A A T C G C G A T G T A C G C T A A G C T G T A C | Fra1(bZIP)/BT549-Fra1-ChIP-Seq(GSE46166)/Homer | 1e-6 | -1.439e+01 | 0.0000 | 143.0 | 11.95% | 3775.0 | 7.87% | motif file (matrix) | svg |
| 56 | T C G A A C G T C A T G G C T A T A G C C G A T G T A C G C T A A C G T A T G C | AP-1(bZIP)/ThioMac-PU.1-ChIP-Seq(GSE21512)/Homer | 1e-5 | -1.381e+01 | 0.0000 | 178.0 | 14.87% | 4994.6 | 10.41% | motif file (matrix) | svg |
| 57 | G C A T C T G A T G C A G A C T A G T C G T A C T C A G C G T A A G C T A C G T G C T A G T A C | Pax7(Paired,Homeobox),long/Myoblast-Pax7-ChIP-Seq(GSE25064)/Homer | 1e-5 | -1.316e+01 | 0.0000 | 20.0 | 1.67% | 226.5 | 0.47% | motif file (matrix) | svg |
| 58 | A T G C T G C A A G T C C G T A A G T C A G T C A C G T A C T G A T G C G T C A | E2A(bHLH),near\_PU.1/Bcell-PU.1-ChIP-Seq(GSE21512)/Homer | 1e-5 | -1.300e+01 | 0.0000 | 255.0 | 21.30% | 7772.6 | 16.20% | motif file (matrix) | svg |
| 59 | C T A G T C G A A C G T A C T G C G T A A T G C A C G T G T A C C G T A A G C T G A T C G T A C | Atf3(bZIP)/GBM-ATF3-ChIP-Seq(GSE33912)/Homer | 1e-5 | -1.248e+01 | 0.0000 | 160.0 | 13.37% | 4488.6 | 9.36% | motif file (matrix) | svg |
| 60 | C T A G T C G A G C A T C A T G G C T A T A G C C G A T G T A C C T G A A G C T | JunB(bZIP)/DendriticCells-Junb-ChIP-Seq(GSE36099)/Homer | 1e-5 | -1.219e+01 | 0.0000 | 134.0 | 11.19% | 3633.0 | 7.57% | motif file (matrix) | svg |
| 61 | T G A C G T A C C G T A A C T G T G A C C G A T A C T G A T C G A G C T T A C G T C G A T A G C G T A C C G T A A T C G T G A C G C A T A C T G A C T G A T G C | Twist(bHLH)/HMLE-TWIST1-ChIP-Seq(Chang\_et\_al)/Homer | 1e-5 | -1.190e+01 | 0.0000 | 37.0 | 3.09% | 656.9 | 1.37% | motif file (matrix) | svg |
| 62 | A T G C T C G A T A C G A C G T A T G C A G T C A C G T A G T C A G T C G A T C | Znf263(Zf)/K562-Znf263-ChIP-Seq(GSE31477)/Homer | 1e-5 | -1.189e+01 | 0.0000 | 257.0 | 21.47% | 7962.7 | 16.60% | motif file (matrix) | svg |
| 63 | T C A G G A C T C A G T C T G A A G C T C T A G G A C T T G C A C T G A A G T C | HLF(bZIP)/HSC-HLF.Flag-ChIP-Seq(GSE69817)/Homer | 1e-5 | -1.181e+01 | 0.0001 | 247.0 | 20.63% | 7611.0 | 15.86% | motif file (matrix) | svg |
| 64 | C T A G A C T G T G C A A G T C C G T A A C T G A C T G A C G T C T A G C G A T T A C G A G T C | ZEB2(Zf)/SNU398-ZEB2-ChIP-Seq(GSE103048)/Homer | 1e-5 | -1.179e+01 | 0.0001 | 171.0 | 14.29% | 4924.4 | 10.26% | motif file (matrix) | svg |
| 65 | T C G A A C T G A C T G C G T A C G T A T C G A A G T C C T G A A T C G G T A C G C A T C A T G | ETS:E-box(ETS,bHLH)/HPC7-Scl-ChIP-Seq(GSE22178)/Homer | 1e-4 | -1.131e+01 | 0.0001 | 29.0 | 2.42% | 468.7 | 0.98% | motif file (matrix) | svg |
| 66 | A G T C G C A T C G T A C G T A G T A C A C G T A C T G G A T C G A T C T C G A | BMYB(HTH)/Hela-BMYB-ChIP-Seq(GSE27030)/Homer | 1e-4 | -1.106e+01 | 0.0001 | 331.0 | 27.65% | 10782.3 | 22.47% | motif file (matrix) | svg |
| 67 | G A C T C A G T A G C T C G A T A G T C G A T C A G T C C G T A A T G C T C A G | Rbpj1(?)/Panc1-Rbpj1-ChIP-Seq(GSE47459)/Homer | 1e-4 | -1.099e+01 | 0.0001 | 285.0 | 23.81% | 9087.6 | 18.94% | motif file (matrix) | svg |
| 68 | G T A C G A T C C A G T A G T C A G T C A G T C T G C A G A T C C T G A A T G C G T C A A C G T | WT1(Zf)/Kidney-WT1-ChIP-Seq(GSE90016)/Homer | 1e-4 | -1.082e+01 | 0.0001 | 80.0 | 6.68% | 1966.1 | 4.10% | motif file (matrix) | svg |
| 69 | C A T G A G T C G A C T C G T A C G A T G C A T G A C T G C A T C G A T C T A G C A T G T G A C | Mef2b(MADS)/HEK293-Mef2b.V5-ChIP-Seq(GSE67450)/Homer | 1e-4 | -1.049e+01 | 0.0002 | 232.0 | 19.38% | 7214.5 | 15.04% | motif file (matrix) | svg |
| 70 | C A G T T G C A A C G T A C T G C G T A A T C G C G A T T G A C C G T A A C G T | BATF(bZIP)/Th17-BATF-ChIP-Seq(GSE39756)/Homer | 1e-4 | -1.038e+01 | 0.0002 | 157.0 | 13.12% | 4566.8 | 9.52% | motif file (matrix) | svg |
| 71 | C T G A C G A T C T A G T C A G G A T C C T G A T C A G G A T C C T G A A C T G A G T C G C T A A C G T A G T C G C A T | PRDM9(Zf)/Testis-DMC1-ChIP-Seq(GSE35498)/Homer | 1e-4 | -1.033e+01 | 0.0002 | 91.0 | 7.60% | 2348.0 | 4.89% | motif file (matrix) | svg |
| 72 | C T A G T C G A A C G T A C T G C G T A T A G C C G A T G T A C C G T A A G C T G A T C G T A C | Jun-AP1(bZIP)/K562-cJun-ChIP-Seq(GSE31477)/Homer | 1e-4 | -1.009e+01 | 0.0003 | 58.0 | 4.85% | 1325.4 | 2.76% | motif file (matrix) | svg |
| 73 | T C G A A C G T A C G T C T G A G A T C T C A G G A C T G T C A C G T A A G C T G T C A C T A G A G C T A C G T T C G A | NFIL3(bZIP)/HepG2-NFIL3-ChIP-Seq(Encode)/Homer | 1e-4 | -1.007e+01 | 0.0003 | 181.0 | 15.12% | 5436.1 | 11.33% | motif file (matrix) | svg |
| 74 | T A C G T A G C G C T A C G A T C T A G A C G T C A G T C A G T G C T A A G T C G T C A G C A T | FOXK2(Forkhead)/U2OS-FOXK2-ChIP-Seq(E-MTAB-2204)/Homer | 1e-4 | -9.795e+00 | 0.0003 | 183.0 | 15.29% | 5535.4 | 11.54% | motif file (matrix) | svg |
| 75 | A T G C G A C T A C G T C T A G A C G T A C G T A C G T C T G A G A T C G C T A A G C T C G T A | Foxa2(Forkhead)/Liver-Foxa2-ChIP-Seq(GSE25694)/Homer | 1e-4 | -9.531e+00 | 0.0004 | 235.0 | 19.63% | 7434.0 | 15.49% | motif file (matrix) | svg |
| 76 | C G A T T A C G T G A C G A C T C A T G C G T A T A C G A C G T G T A C C T G A | Bach2(bZIP)/OCILy7-Bach2-ChIP-Seq(GSE44420)/Homer | 1e-4 | -9.290e+00 | 0.0005 | 51.0 | 4.26% | 1153.8 | 2.40% | motif file (matrix) | svg |
| 77 | G A C T C T A G C T A G A G T C T G C A A C T G A C G T A C G T C T A G T C A G | AMYB(HTH)/Testes-AMYB-ChIP-Seq(GSE44588)/Homer | 1e-4 | -9.218e+00 | 0.0006 | 322.0 | 26.90% | 10694.6 | 22.29% | motif file (matrix) | svg |
| 78 | C T A G T C G A C G A T A C T G C G T A T A C G A G C T T G A C G C T A A C G T G A T C T A G C | Fosl2(bZIP)/3T3L1-Fosl2-ChIP-Seq(GSE56872)/Homer | 1e-3 | -9.190e+00 | 0.0006 | 75.0 | 6.27% | 1906.1 | 3.97% | motif file (matrix) | svg |
| 79 | G C A T C G T A C G A T A C T G A G T C G C T A C T G A C G T A C A G T A C T G C G T A T C A G | Oct6(POU,Homeobox)/NPC-Pou3f1-ChIP-Seq(GSE35496)/Homer | 1e-3 | -9.145e+00 | 0.0006 | 147.0 | 12.28% | 4334.3 | 9.03% | motif file (matrix) | svg |
| 80 | C A T G A G T C A G C T C G T A C G A T C G A T G C A T G C A T C G A T C T G A C A T G T G A C | Mef2d(MADS)/Retina-Mef2d-ChIP-Seq(GSE61391)/Homer | 1e-3 | -8.176e+00 | 0.0015 | 63.0 | 5.26% | 1586.3 | 3.31% | motif file (matrix) | svg |
| 81 | C A G T T C A G A G C T G A C T A C G T A G T C G A T C G A C T C T G A A C T G G A T C C G T A C T G A A G T C G T A C | Rfx6(HTH)/Min6b1-Rfx6.HA-ChIP-Seq(GSE62844)/Homer | 1e-3 | -7.446e+00 | 0.0032 | 193.0 | 16.12% | 6164.8 | 12.85% | motif file (matrix) | svg |
| 82 | C A G T T C A G G A T C A C T G A C G T C T A G A C T G A C T G G A C T C T A G | Egr1(Zf)/K562-Egr1-ChIP-Seq(GSE32465)/Homer | 1e-3 | -7.124e+00 | 0.0043 | 82.0 | 6.85% | 2284.7 | 4.76% | motif file (matrix) | svg |
| 83 | C T A G T C G A C G A T C T A G G C A T C A G T C T A G G A T C C G T A G T C A | CEBP:AP1(bZIP)/ThioMac-CEBPb-ChIP-Seq(GSE21512)/Homer | 1e-3 | -7.090e+00 | 0.0044 | 186.0 | 15.54% | 5955.7 | 12.41% | motif file (matrix) | svg |
| 84 | T A G C T A G C G A C T C T A G A G C T A G T C G T C A T G C A A C G T A T G C G C T A T G C A | Pbx3(Homeobox)/GM12878-PBX3-ChIP-Seq(GSE32465)/Homer | 1e-3 | -7.033e+00 | 0.0046 | 55.0 | 4.59% | 1402.2 | 2.92% | motif file (matrix) | svg |
| 85 | G A C T C A G T G A T C G A T C A C G T G A T C C T G A T A C G C G T A G T C A | STAT6(Stat)/Macrophage-Stat6-ChIP-Seq(GSE38377)/Homer | 1e-3 | -6.929e+00 | 0.0051 | 139.0 | 11.61% | 4282.5 | 8.93% | motif file (matrix) | svg |
| 86 | G A C T C G T A A C G T A C T G A G T C C G T A C T G A C G T A C A G T A C G T G T C A T C A G | Brn1(POU,Homeobox)/NPC-Brn1-ChIP-Seq(GSE35496)/Homer | 1e-2 | -6.766e+00 | 0.0059 | 105.0 | 8.77% | 3103.5 | 6.47% | motif file (matrix) | svg |
| 87 | C G T A G C T A C G A T C T A G A C G T G T C A C G T A C G T A A G T C C G T A T G C A T A C G | FoxL2(Forkhead)/Ovary-FoxL2-ChIP-Seq(GSE60858)/Homer | 1e-2 | -6.584e+00 | 0.0070 | 278.0 | 23.22% | 9442.5 | 19.68% | motif file (matrix) | svg |
| 88 | T A C G C T A G T A C G A G T C C G T A A G T C A G T C A C G T A C T G A G T C G A T C T A G C | Slug(Zf)/Mesoderm-Snai2-ChIP-Seq(GSE61475)/Homer | 1e-2 | -6.305e+00 | 0.0091 | 90.0 | 7.52% | 2628.0 | 5.48% | motif file (matrix) | svg |
| 89 | T C A G G C T A T C A G C A G T T G A C G T C A A G T C A T C G T G C A G T A C C A G T G A T C | Npas4(bHLH)/Neuron-Npas4-ChIP-Seq(GSE127793)/Homer | 1e-2 | -6.075e+00 | 0.0114 | 110.0 | 9.19% | 3349.7 | 6.98% | motif file (matrix) | svg |
| 90 | T A C G C T G A T C G A C G A T C T A G C T A G T C G A C T G A T C G A T C G A C G T A T C G A G C A T C A T G C G T A T A C G G C A T T G A C C G T A A G C T | NFAT:AP1(RHD,bZIP)/Jurkat-NFATC1-ChIP-Seq(Jolma\_et\_al.)/Homer | 1e-2 | -5.960e+00 | 0.0126 | 47.0 | 3.93% | 1214.4 | 2.53% | motif file (matrix) | svg |
| 91 | A T G C G A T C C G T A A G C T C A G T A T C G G C A T A G C T G A C T A C T G | Sox17(HMG)/Endoderm-Sox17-ChIP-Seq(GSE61475)/Homer | 1e-2 | -5.910e+00 | 0.0131 | 186.0 | 15.54% | 6117.1 | 12.75% | motif file (matrix) | svg |
| 92 | T G C A A G C T C A T G C G T A A G C T A C T G G A T C G T C A C G T A A G C T | Atf4(bZIP)/MEF-Atf4-ChIP-Seq(GSE35681)/Homer | 1e-2 | -5.868e+00 | 0.0135 | 72.0 | 6.02% | 2051.2 | 4.28% | motif file (matrix) | svg |
| 93 | G T A C C G T A C G T A T A C G G C A T G T A C C G T A C A T G A G T C C G T A C G T A C G A T G C A T G C A T G A C T | MafF(bZIP)/HepG2-MafF-ChIP-Seq(GSE31477)/Homer | 1e-2 | -5.501e+00 | 0.0193 | 83.0 | 6.93% | 2463.1 | 5.13% | motif file (matrix) | svg |
| 94 | C G A T C T A G C T G A A T G C C T G A T C G A C G T A C T G A T C G A T A G C A G T C C G T A A C T G T C G A A T G C | Hand2(bHLH)/Mesoderm-Hand2-ChIP-Seq(GSE61475)/Homer | 1e-2 | -5.331e+00 | 0.0227 | 96.0 | 8.02% | 2936.0 | 6.12% | motif file (matrix) | svg |
| 95 | A G T C G T A C A G C T C T A G A G T C C G A T A C T G C G T A A C T G G T C A | Zic(Zf)/Cerebellum-ZIC1.2-ChIP-Seq(GSE60731)/Homer | 1e-2 | -5.242e+00 | 0.0245 | 136.0 | 11.36% | 4381.1 | 9.13% | motif file (matrix) | svg |
| 96 | A T G C G A T C C G A T A C G T A C G T A C T G C A G T A G C T | Sox3(HMG)/NPC-Sox3-ChIP-Seq(GSE33059)/Homer | 1e-2 | -5.028e+00 | 0.0300 | 406.0 | 33.92% | 14656.3 | 30.55% | motif file (matrix) | svg |
| 97 | A G T C G A T C G C T A C G A T A C G T T A C G G C A T C T G A G A C T A C T G A G T C G C T A C T G A T C G A C A G T | Oct4:Sox17(POU,Homeobox,HMG)/F9-Sox17-ChIP-Seq(GSE44553)/Homer | 1e-2 | -5.023e+00 | 0.0300 | 50.0 | 4.18% | 1379.5 | 2.88% | motif file (matrix) | svg |
| 98 | G C A T G C A T C T G A A C G T C T G A A C G T C G T A C G T A C G T A A G T C G T C A G T C A | Foxf1(Forkhead)/Lung-Foxf1-ChIP-Seq(GSE77951)/Homer | 1e-2 | -5.012e+00 | 0.0300 | 296.0 | 24.73% | 10411.5 | 21.70% | motif file (matrix) | svg |
| 99 | A G C T A T G C G A C T G C A T C G T A A G C T G T A C C G A T A T C G A G T C | Gata6(Zf)/HUG1N-GATA6-ChIP-Seq(GSE51936)/Homer | 1e-2 | -4.929e+00 | 0.0321 | 232.0 | 19.38% | 7994.9 | 16.66% | motif file (matrix) | svg |
| 100 | T A C G A T G C G A C T A C T G A G C T A G T C G T C A T G C A A C G T A G T C G C T A T G C A | Pknox1(Homeobox)/ES-Prep1-ChIP-Seq(GSE63282)/Homer | 1e-2 | -4.795e+00 | 0.0364 | 45.0 | 3.76% | 1230.7 | 2.57% | motif file (matrix) | svg |
| 101 | C A T G G T A C G A C T G C T A C G T A C G T A C G T A G C T A G A C T C T G A T C A G G T A C | Mef2c(MADS)/GM12878-Mef2c-ChIP-Seq(GSE32465)/Homer | 1e-2 | -4.770e+00 | 0.0369 | 127.0 | 10.61% | 4118.1 | 8.58% | motif file (matrix) | svg |
| 102 | C A T G T A C G T A G C G A T C G A T C A T G C G T A C G A C T T C A G A T G C C G A T A T C G C A G T A C T G G T A C | Zic3(Zf)/mES-Zic3-ChIP-Seq(GSE37889)/Homer | 1e-2 | -4.649e+00 | 0.0413 | 65.0 | 5.43% | 1922.7 | 4.01% | motif file (matrix) | svg |
| 103 | A G C T A G T C A T G C A G C T A C G T C G T A A C G T A G T C C G A T A T G C | Gata2(Zf)/K562-GATA2-ChIP-Seq(GSE18829)/Homer | 1e-2 | -4.644e+00 | 0.0413 | 158.0 | 13.20% | 5275.1 | 10.99% | motif file (matrix) | svg |
| 104 | T A C G A T C G G A T C G T C A C T G A G C A T C G A T G C T A T C G A G C T A | Unknown(Homeobox)/Limb-p300-ChIP-Seq/Homer | 1e-2 | -4.607e+00 | 0.0422 | 189.0 | 15.79% | 6435.7 | 13.41% | motif file (matrix) | svg |
